# Phenotypic heterogeneity in m.3243A>G mitochondrial disease: The role of nuclear factors

**DOI:** 10.1002/acn3.532

**Published:** 2018-02-07

**Authors:** Sarah J. Pickett, John P. Grady, Yi Shiau Ng, Gráinne S. Gorman, Andrew M. Schaefer, Ian J. Wilson, Heather J. Cordell, Doug M. Turnbull, Robert W. Taylor, Robert McFarland

**Affiliations:** ^1^ Wellcome Centre for Mitochondrial Research Institute of Neuroscience Newcastle University Newcastle upon Tyne UK; ^2^ Institute of Genetic Medicine Newcastle University Newcastle upon Tyne UK; ^3^ Present address: Kinghorn Centre for Clinical Genomics Garvan Institute Sydney NSW Australia

**Keywords:** mitochondrial disease, m.3243A>G, heritability

## Abstract

**Objective:**

The pathogenic mitochondrial DNA m.3243A>G mutation is associated with a wide range of clinical features, making disease prognosis extremely difficult to predict. We aimed to understand the cause of this heterogeneity.

**Methods:**

We examined the phenotypic profile of 238 adult m.3243A>G carriers (patients and asymptomatic carriers) from the UK MRC Mitochondrial Disease Patient Cohort using the Newcastle Mitochondrial Disease Adult Scale. We modeled the role of risk factors for the development of specific phenotypes using proportional odds logistic regression. As mitochondria are under the dual control of their own and the nuclear genome, we examined the role of additive nuclear genetic factors in the development of these phenotypes within 46 pedigrees from the cohort.

**Results:**

Seizures and stroke‐like episodes affect 25% and 17% of patients, respectively; more common features include hearing impairment, gastrointestinal disturbance, psychiatric involvement, and ataxia. Age, age‐adjusted blood heteroplasmy levels, and sex are poor predictors of phenotypic severity. Hearing impairment, diabetes, and encephalopathy show the strongest associations, but pseudo‐*R* ^2^ values are low (0.14–0.17). We found a high heritability estimate for psychiatric involvement (*h* ^2^=0.76, *P* = 0.0003) and moderate estimates for cognition (*h* ^2^=0.46, *P* = 0.0021), ataxia (*h* ^2^ = 0.45, *P* = 0.0011), migraine (*h* ^2^ = 0.41, *P* = 0.0138), and hearing impairment (h^2^ = 0.40, *P* = 0.0050).

**Interpretation:**

Our results provide good evidence for the presence of nuclear genetic factors influencing clinical outcomes in m.3234A>G‐related disease, paving the way for future work identifying these through large‐scale genetic linkage and association studies, increasing our understanding of the pathogenicity of m.3243A>G and providing improved estimates of prognosis.

## Introduction

Mitochondria, essential organelles responsible for oxidative phosphorylation (OXPHOS), are under the dual control of their own and the nuclear genome. Mitochondrial DNA (mtDNA) encodes 13 subunits of the OXPHOS system, along with 22 mitochondrial tRNAs and two mitoribosomal subunits.^1^ An additional ~1150 proteins are nuclear‐encoded, including further OXPHOS subunits and proteins related to other essential mitochondrial functions.^2^ Mutations in nuclear and mtDNA are responsible for a clinically heterogeneous group of mitochondrial diseases.^3^

To explore the cause of this heterogeneity, we studied the pathogenic m.3243A>G mtDNA variant within *MT‐TL1* (encoding mitochondrial tRNA^Leu(UUR)^), which represents the most common heteroplasmic mtDNA disease genotype.^3^, ^4^, ^5^, ^6^ About 15% m.3243A>G patients present with mitochondrial encephalopathy, lactic acidosis, and stroke‐like episodes (MELAS) syndrome; the remainder display a wide variety of clinical features including diabetes, deafness, ataxia, and isolated myopathy.^7^ Population‐based studies estimate a carrier frequency of 140–250 per 100,000, whereas the estimated disease prevalence is 40–70 times lower.^5^, ^6^, ^8^ Therefore, many carriers are clinically asymptomatic or have mild disease not recognized as mitochondrial.

Heteroplasmy describes the coexistence of mutant and wild‐type mtDNA molecules within the same cell. mtDNA mutant levels can vary dramatically between different individuals and tissues; postmitotic tissues such as muscle have higher levels, whilst levels in blood decrease significantly over time.^4^, ^9^, ^10^, ^11^ Heteroplasmy contributes to, but does not fully explain, the observed heterogeneity.^12^, ^13^, ^14^ Patients with identical tissue m.3243A>G heteroplasmy levels can exhibit very different symptoms, and some patients with high heteroplasmy levels are relatively asymptomatic. Disease progression in patients with single, large‐scale mtDNA deletions can be predicted using percentage mutation load, deletion size, and genomic position.^15^ However, heteroplasmy and age are only weakly correlated with m.3243A>G‐related disease severity and cannot be used to predict key clinical outcomes such as stroke‐like episodes.^15^ Therefore, counseling patients regarding their likely prognosis and female carriers regarding risk to future offspring is enormously problematic.

Such unexplained heterogeneity suggests that unidentified factors influence phenotype; the nuclear genome is a strong candidate given the interaction between the two genomes. Nuclear background has been shown to influence mitochondrial function, with a mouse model showing decreased fitness and lifespan when mitochondrial and nuclear mutations are combined.^16^ A recent report describing two pairs of monozygotic twins highlighted a strikingly similar clinical phenotype, age‐of‐onset, and tissue m.3243A>G heteroplasmy levels in each pair.^17^ Furthermore, there is evidence for the presence of nuclear modifiers in other maternally inherited mtDNA mutations in humans^18^ and the control of mtDNA segregation in mice.^19^

The extent to which genetic factors contribute to a phenotype can be assessed using familial relationships to estimate heritability.^20^ This represents the proportion of trait variance that can be attributed to inherited genetic factors, therefore prioritizing traits for further genetic analysis.

We aimed to model the development of specific m.3243A>G‐related phenotypes within a large UK patient cohort to allow the prediction of disease prognosis in patients. We examined the role of age, heteroplasmy, and sex as risk factors and evaluated the potential role of additive nuclear genetic factors by exploiting familial relationships to generate estimates of heritability.

## Materials and methods

### Study population

These studies were undertaken using data from 238 m.3243A>G carriers from the MRC Mitochondrial Disease Patient Cohort UK.^7^ All individuals were reviewed and investigated by the NHS Highly Specialised Service for Rare Mitochondrial Disorders of Adults and Children in Newcastle upon Tyne. Patient follow‐up was carried out (median interval = 1.11 years, IQR = 0.73) using the Newcastle Mitochondrial Disease Adult Scale (NMDAS), a validated scale to evaluate multisystem involvement and disease burden in adult mitochondrial disease.^22^ Recruitment is ongoing; here we describe the current phenotypic profile of the cohort, which displays a wide spectrum of disease (median total NMDAS = 19.5, IQR = 25.9, range = 0–104). In our analyses, we have included 202 symptomatic patients (total NMDAS score ≥5), 32 relatively asymptomatic carriers (NMDAS <5 with a detectable m.3243A>G mutation), and 4 with incomplete NMDAS data.

The median age at last assessment was 43.4 years (IQR = 23.2, range = 15.1–79.9), and only two individuals were <18 years old. For the patients with multiple NMDAS assessments (*N* = 175), the median follow‐up time was 3.60 years (IQR = 4.99, range = 0.05–11.90) with a median of 4 assessments per patient (IQR = 5, max 17).

NMDAS and blood m.3243A>G heteroplasmy data were available for 219 subjects (132 females); 157 of these were from families with multiple members within the cohort, comprising 46 familial lineages with multiple members, and 62 individuals who had no relatives in the cohort. Pedigrees range in size from two to seven individuals (median 3, IQR = 2), and span 2–4 generations.

Ethical approval was granted by the Newcastle and North Tyneside Research Ethics Committee (13/NE/0326), and written informed consent from patients was obtained prior to study inclusion. All clinical investigations were evaluated according to the Declaration of Helsinki.

### Phenotypes

Nineteen questions from the NMDAS (http://www.newcastle-mitochondria.com/clinical-professional-home-page/clinical-publications/nmdas/) were considered. Section I of NMDAS scores current function according to patients and/or caregiver interview and relates to the preceding 4 weeks. Most questions from this NMDAS section were omitted from these analyses due to their subjective nature. The exception was hearing impairment which was included as it is one of the common symptoms for m.3243A>G‐related disease, and is the only question that captures information about hearing. All questions are independently assessed; therefore, the exclusion of questions does not affect the validity of the sections that were retained in the study. Section II scores system specific involvement according to patients and/or caregiver interview and consultation with medical notes and relates to the preceding 12‐month period and section III scores current clinical assessment according to an examination performed at the time of assessment; both are likely to be more objective and so all were included. Some traits, such as stroke‐like episodes, can be stochastic in nature; individuals can score very low on one assessment despite historically high scores. Therefore, to ensure we captured these historical events, a single independent point for each trait and each individual was determined by the maximal NMDAS score and the youngest age at which that score was attained. For asymptomatic individuals, the age at the most recent assessment was used.

### Heteroplasmy measurements

Total DNA was extracted from whole blood by standard procedures. Pyrosequencing on the Pyromark Q24 platform permitted quantification of m.3243A>G heteroplasmy levels, as previously described and validated,^22^ with mutation‐specific pyrosequencing primers according to GenBank Accession number NC_012920.1: 5′biotinylated forward: m.3143‐3163; reverse: m.3331‐3353; and reverse pyrosequencing primer: m.3244‐3258 (IDT, Coralville). The allele quantification application of Pyromark's proprietary Q24 software was used to calculate heteroplasmy levels (level of test sensitivity >3% mutant mtDNA).

In blood, the m.3243A>G heteroplasmy level progressively declines by ~2.3% year^−1^ and recent work has shown that age‐corrected blood heteroplasmy is a better predictor for disease progression than mean urine heteroplasmy levels and sex‐corrected mean urine heteroplasmy levels (unpublished data). Given this exponential decline, m.3243A>G levels were corrected for age using the following formula: Age‐adjusted blood level = Blood heteroplasmy/0.977^(age+12)^ The median age‐adjusted blood heteroplasmy level was 60.1% (IQR = 53.9, range = 2.1–100).

### Statistical analysis

Pearson correlations between traits and significance levels were calculated using the rcorr function in the Hmisc package in R.^24^, ^25^

Specific phenotypic feature analysis used proportional odds multiple logistic regression, with age, age‐adjusted mean blood heteroplasmy, and sex as predictors, using clm from the ordinal package in R.^23^, ^25^ Sex was dropped from the model when the significance threshold (*P* = 0.05, determined by maximum likelihood testing) was not reached. This was the case for all phenotypes except ptosis, myopathy, and gastrointestinal disturbance. NMDAS scores were re‐categorized as asymptomatic/mild (NMDAS = 0–1), moderate (2–3), and severe (4–5) to ensure sufficient patients in each group for statistical modeling.

Assumptions of the proportional odds model were tested by comparing fitted models with models where the effects were modeled as nominal rather than ordinal, using a likelihood ratio test and a significance threshold of *P* = 0.05. Encephalopathy did not meet the assumption of proportional odds so was considered as a binary trait using threshold scores determined a priori based on the NMDAS clinical descriptors,^21^ using clinical judgement and ensuring a minimum of 30 subjects in the affected group (Table 1). Due to small numbers, extrapyramidal involvement was also considered as a binary trait with moderate and severe scores (NMDAS = 2–5) combined into one category.

**Table 1:** NMDAS traits including threshold values for analysis as binary traits

Trait	Threshold score	Phenotype description	NMDAS section
Psychiatric involvement	3	Moderate to severe, warranting specialist treatment	II
Cerebellar ataxia	2	At least unable to maintain heel‐toe walking or displaying mild unilateral dysmetria	III
Migraine	5	Four days per month or more	II
Cognition	3	Combined centiles of 29 or below on Wechsler Test of Adult Reading, Symbol Search and Speed of Comprehension Test	III
Neuropathy	2	At least sensory impairment (e.g., glove and stocking sensory loss)	III
Dysphonia‐dysarthria	2	Showing at least mild impairment (clear impairment but easily understood)	III
Seizures	1	At least past history of epilepsy	II
Encephalopathy	1	At least past history of encephalopathy	II
Stroke‐like episodes	1	At least transient focal sensory symptoms	II
Visual acuity	3	Combined Snellen fraction > 6/36	III
Ptosis	2	At least unilateral ptosis obscuring 1/3 of pupil	III
CPEO	2	Abduction of at least one eye incomplete	III
Hearing impairment	3	At least moderate deafness (e.g. regularly requiring repetition), not fully corrected with hearing aid	I
Gastrointestinal disturbance	4	Dysmotility requiring admission or persistent and/or recurrent anorexia/vomiting/weight loss	II
Myopathy	2	Mild but clear proximal weakness in hip flexion and shoulder abduction and minimal weakness in elbow flexion and knee extension	III
Diabetes	2	At least impaired glucose tolerance (in absence of intercurrent illness)	II
Cardiovascular involvement	2	At least asymptomatic left ventricular hypertrophy on echo or nonsustained brady/tachyarrhythmia on ECG	II

Thresholds were defined based on the NMDAS clinical descriptors for each trait, to ensure traits were as objective as possible.

We report McFadden's pseudo‐*R* ^2^ values,^26^ which estimate the proportion of observed variance described by the fitted model. This is defined by 1−log(L_c_)/log(L_null_), where L_c_ is the maximum likelihood of the fitted model and L_null_ is the maximum likelihood of the null.

Odds ratios (relating to decades for age and 10% change in level for heteroplasmy) were calculated by taking exponentials of the coefficients estimated by the model. They represent the odds that an individual will move from one category to the next (asymptomatic/mild to moderate or moderate to severe) given an increase of a decade (age) or 10% (heteroplasmy) in the predictor variables, compared to the odds in the absence of an increase.

Unless otherwise stated, we report unadjusted *P* values as for reasons well documented in the literature, Bonferroni correction would be too conservative, particularly as we are testing a priori hypotheses with variables that are not all independent.^27^

### Calculation of heritability

Heritability in the narrow sense (*h* ^2^) is the proportion of the total population variance of a trait ($\sigma_{p}^{2}$) that can be attributed to additive nuclear genetic effects ($\sigma_{a}^{2}$), expressed as a ratio ($h^{2}=\sigma_{a}^{2}/\sigma_{p}^{2}$). Heritability was estimated using variance components methods and significance levels obtained using likelihood ratio tests, as implemented in the SOLAR software package (SOLAR Eclipse version 8.1.1).^28^ Results were corrected for ascertainment bias by conditioning on proband status, as implemented in SOLAR. This information was available for 38 pedigrees; data from probands in the remaining 14 pedigrees were not available due to death before the study began.

To produce heritability estimates which excluded the variation due to known factors such as age and heteroplasmy level, we included these factors as covariates in these analyses. Sex was included where it was shown to have an effect at the *P* < 0.1 level (migraine, dysphonia‐dysarthria, ptosis, gastrointestinal disturbance, and myopathy). The square root of trait score was used for cerebellar ataxia, neuropathy, dysphonia–dysarthria, encephalopathy, stroke‐like episodes, visual acuity, and chronic progressive external ophthalmoplegia (CPEO), in order to ensure normality of the residuals (kurtosis <0.8). After this transformation, only stroke‐like episodes and encephalopathy showed residual kurtosis outside of the acceptable range, and this is likely to be due to the small number of affected individuals for these traits. Pyramidal and extrapyramidal involvements were excluded from the heritability analysis due to low numbers of affected individuals.

For binary trait analysis, individuals with a score greater than or equal to a threshold score (Table 1) were coded as affected, all others as unaffected. For stroke‐like episodes, only those patients with radiologically confirmed stroke‐like episodes were coded as affected. We report heritabilities (*h* ^2^), standard error (SE), P values, and Kullback‐Leibler *R* ^2^, as calculated in SOLAR.^28^ Models for neuropathy, seizures, gastrointestinal disturbance, and myopathy did not converge and so are not presented.

To determine whether common genetic factors influence pairs of traits, bivariate heritability analysis was performed. SOLAR calculates the estimated genetic correlation (rhoG), a measure of the shared genetic variance between traits (pleiotropy). Significant (determined by likelihood ratio test) genetic correlations suggest the presence of common genetic factors (one or more genes) that influence both traits.^29^

## Results

### Marked clinical variation in the phenotypic profile of the m.3243A>G cohort

**Figure 1:**
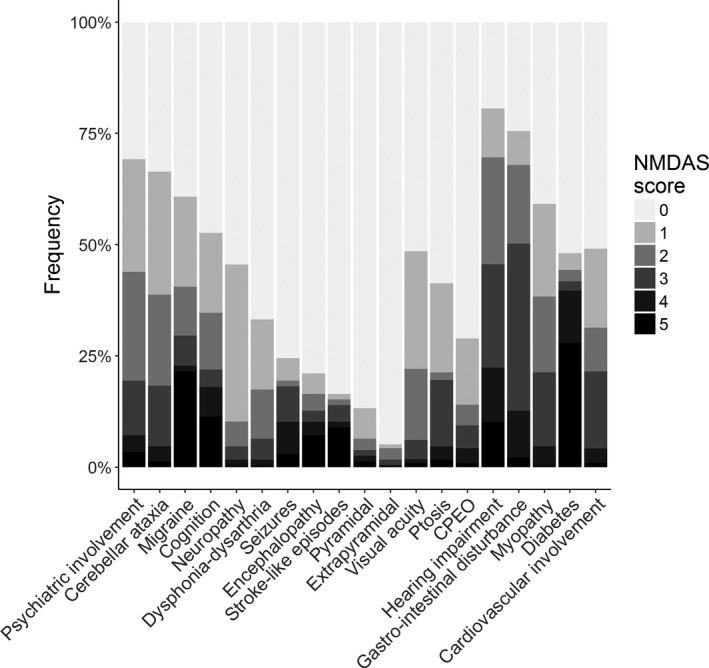
Phenotypic profile of the cohort. For each trait, the maximum score attained by each patient in any NMDAS assessment was noted. The total number of patients assessed for each trait ranges from 214 to 237.

First, we examined the range of clinical phenotypes observed in our patient cohort, as assessed by the NMDAS. The most common phenotypic traits associated with the pathogenic m.3243A>G variant are hearing impairment (81%; NMDAS≥1) and gastrointestinal disturbance (76%), followed by psychiatric involvement (69%) and cerebellar ataxia (66%) (Fig. 1). Half of our patients exhibit mild psychiatric involvement (e.g., reactive depression, NMDAS=1/2), but 19% demonstrate a moderate to severe disorder (NMDAS=3/4/5). Seizures, encephalopathy, and stroke‐like episodes affect 25%, 21%, and 17% of patients respectfully. Both pyramidal and extrapyramidal involvements are rare in the cohort at 13% and 5% respectively. We also inspected the distribution of NMDAS scores (Fig. 1). For hearing impairment, the distribution is reasonably uniform, in comparison with cerebellar ataxia where each successive level of severity is progressively rarer in the cohort. Diabetes and stroke‐like episodes demonstrate bi‐modal distributions, with patients generally either asymptomatic or severely affected.

### A number of phenotypic traits are correlated

**Figure 2:**
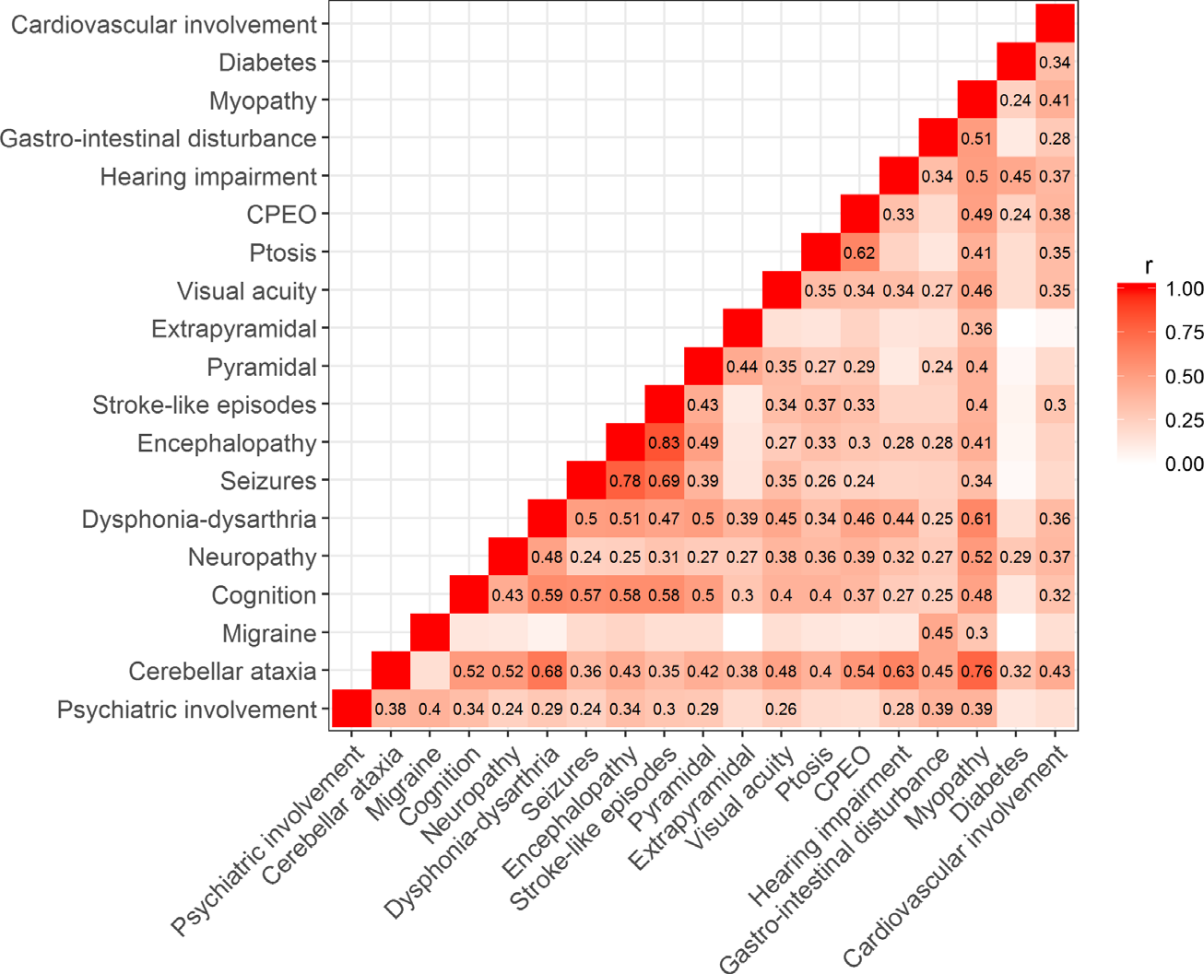
Heat map showing correlations between trait scores. Estimated Pearson correlation coefficients (r) are shown where the test reached a Bonferroni adjusted significance threshold of *P* = 0.00029 (0.05/171).

We then asked whether traits commonly observed in association with the pathogenic m.3243A>G variant are correlated with each other, which would provide evidence of a common underlying etiology (Fig. 2). Unsurprisingly, seizures, encephalopathy, and stroke‐like episodes are well correlated (estimated Pearson correlation coefficient (r) range ~ 0.69–0.83) and so are ptosis and CPEO (*r* ~ 0.62). Hearing impairment and diabetes are moderately correlated (*r* ~ 0.45), confirming a well‐reported association between these two traits.^30^ Cognition shows moderate to strong correlations with other CNS traits, such as dysphonia–dysarthria, seizures, stroke‐like episodes, encephalopathy, seizures, and cerebellar ataxia (*r* ~ 0.52–0.59). Cerebellar ataxia is correlated with a number of traits, notably myopathy (*r* ~ 0.76), hearing impairment (*r* ~ 0.63), and dysphonia–dysarthria (*r* ~ 0.68).

Interestingly, both migraine and diabetes are only correlated with a small number of other traits. Migraine is, however, moderately correlated with gastrointestinal disturbance (*r* ~ 0.45).

### Age, heteroplasmy, and sex are poor predictors of phenotypic severity

**Figure 3:**
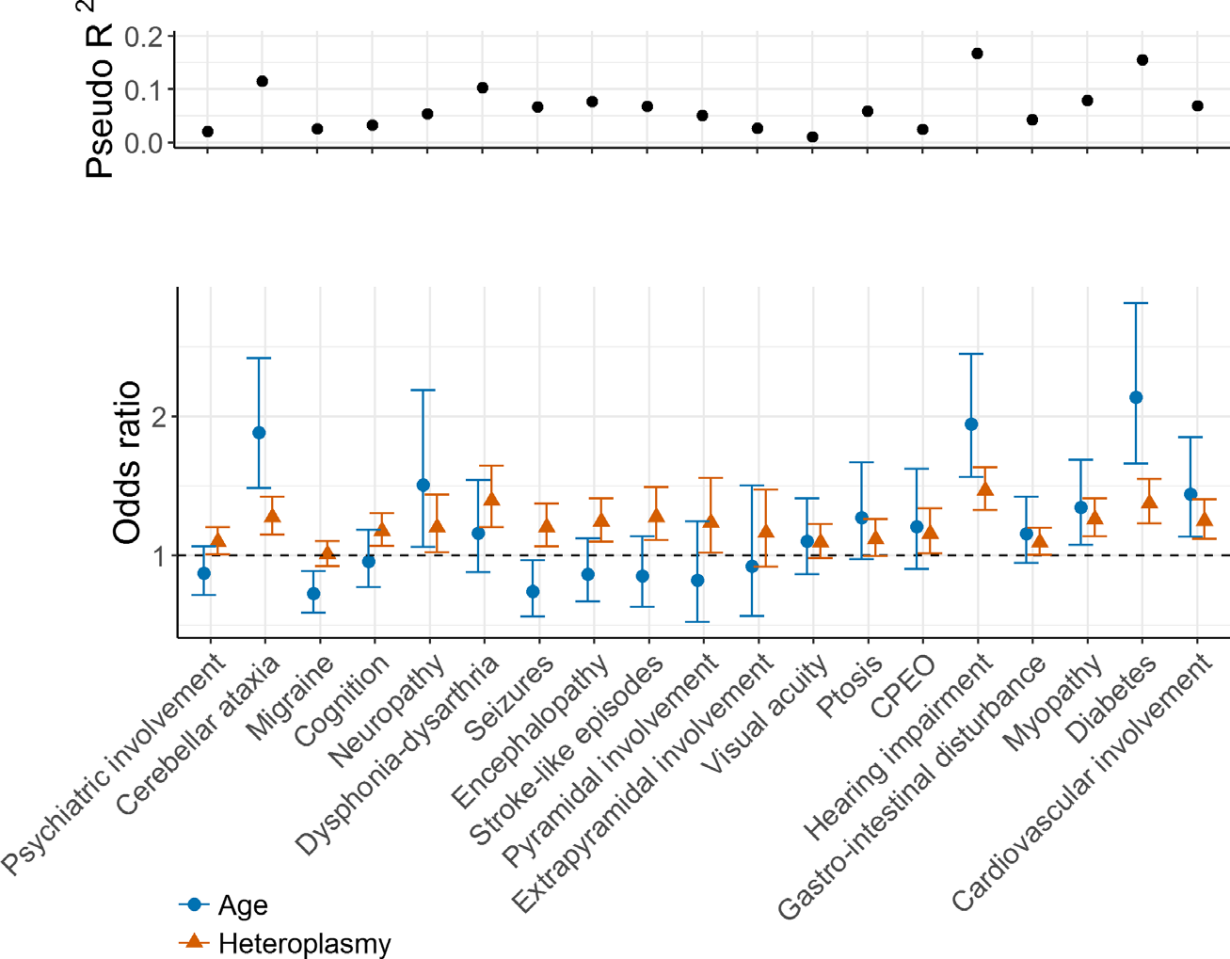
McFadden's pseudo‐*R* ^2^ values and odds ratios (with 95% confidence intervals) showing the effect of age and heteroplasmy level (decades for age and 10% change in level for heteroplasmy) on the risk of developing specific m.3243A>G‐related disease traits. Odds ratios with confidence intervals that do not cross the dotted line at OR = 1 are significant at the *P* < 0.05 level. The total number of patients included in the analyses ranges from 193 to 212.

Next, we explored the impact of age, heteroplasmy level, and sex on the severity of specific phenotypic features using proportional odds logistic regression (Fig. 3). Odds ratios (ORs; relating to decades for age and 10% change in level for heteroplasmy) show the clinical effect size of each factor. Higher severity of diabetes (OR = 2.14, *P* = 1.5 × 10^−8^), hearing impairment (OR = 1.95, *P* = 4.7 × 10^−9^), cerebellar ataxia (OR = 1.88, *P* = 3.2 × 10^−7^), and neuropathy (OR = 1.51, *P* = 2.5 × 10^−2^) are most strongly associated with increasing age, consistent with the clinical picture of a progressive disease. Almost all features were significantly associated with higher heteroplasmy, the most strongly associated being hearing impairment (OR = 1.47, *P* = 7.2 × 10^−13^), dysphonia–dysarthria (OR = 1.39, *P* = 2.8 × 10^−5^), diabetes (OR = 1.37, *P* = 5.7 × 10^−8^), stroke‐like episodes (OR = 1.28, *P* = 1.0 × 10^−3^), cerebellar ataxia (OR = 1.27, *P* = 8.6 × 10^−6^), myopathy (OR = 1.26, *P* = 2.2 × 10^−5^), cardiovascular involvement (OR = 1.25, *P* = 1.0 × 10^−4^), and encephalopathy (OR = 1.24, *P* = 6.0 × 10^−4^). Although McFadden's pseudo‐*R* ^2^ values indicate that hearing impairment (0.17, *P* = 2.5 × 10^−17^), diabetes (0.16, *P* = 4.1 × 10^−13^), and cerebellar ataxia (0.12, *P* = 1.8 × 10^−9^) show the strongest association between higher phenotypic severity, higher age‐adjusted blood heteroplasmy, and increasing age, pseudo‐*R* ^2^ values are low, highlighting the limited predictive power of these covariates.

Several key features of the MELAS phenotype, including stroke‐like episodes, seizures, and encephalopathy, were not associated with age, only with heteroplasmy. This is consistent with clinical observations.

For two phenotypes, the effect of sex was significant (ptosis; OR_males_ = 3.02, *P* = 2.6 × 10^−3^ and myopathy; OR_males_ = 0.43, *P* = 7.9 × 10^−3^). Although more males (17/95, 17.9%) were affected by stroke‐like episodes than females (15/144, 10.4%), this did not reach significance (Fisher's exact test, *P* = 0.12).

### m.3243A>G‐related phenotypes are heritable

As low pseudo‐*R* ^2^ values indicate that a large proportion of the phenotypic variance remains to be explained, we then explored the likelihood of nuclear genetic factors contributing to phenotypic variance in our cohort by calculating heritability (*h* ^2^) of NMDAS traits using the information contained within pedigree structures.

We found a significant, high heritability estimate for psychiatric involvement (*h* ^2^=0.76, *P* = 0.0003) and moderate estimates for cognition (*h* ^2^ = 0.46, *P* = 0.0021), cerebellar ataxia (*h* ^2^ = 0.45, *P* = 0.0011), migraine (*h* ^2^=0.41, *P* = 0.0138), and hearing impairment (*h* ^2^=0.40, *P* = 0.0050) (Table 2). Diabetes, visual acuity, and encephalopathy have estimates that are close to significance (*P* < 0.08). These results, which take age‐adjusted blood heteroplasmy, age, and sex into account, provide good evidence for the presence of nuclear genetic factors influencing several clinical outcomes in m.3234A>G‐related disease.

**Table 2:** Estimates of the proportion of trait variance due to known covariates (age‐adjusted blood heteroplasmy, age, and sex) and additive genetic factors (heritability)

Trait	Number of cases	*h* ^2^	SE	*P* value	Proportion of variance due to covariates
Psychiatric involvement	**214**	**0.76**	**0.21**	**0.0003**	**0.03**
Cerebellar ataxia^b^	**213**	**0.45**	**0.16**	**0.0011**	**0.40**
Migraine	**214**	**0.41**	**0.19**	**0.0138**	**0.04** a
Cognition	**205**	**0.46**	**0.17**	**0.0021**	**0.06**
Neuropathy^b^	213	0.14	0.19	0.2257	0.09
Dysphonia–dysarthria^b^	213	0.18	0.15	0.0992	0.18^a^
Seizures^b^	214	0.05	0.13	0.3605	0.10
Encephalopathy^b^	214	0.18	0.12	0.0624	0.08
Stroke‐like episodes^b^	214	0.12	0.13	0.1509	0.07
Visual acuity^b^	210	0.31	0.21	0.0570	0.06
Ptosis	213	0.20	0.21	0.1489	0.11^a^
CPEO^b^	213	0.28	0.22	0.0926	0.11
Hearing impairment	**214**	**0.40**	**0.16**	**0.0050**	**0.36**
Gastrointestinal disturbance	214	0.06	0.19	0.3770	0.13^a^
Myopathy	213	0.07	0.17	0.3350	0.19^a^
Diabetes	214	0.28	0.19	0.0715	0.27
Cardiovascular involvement	193	0.09	0.19	0.3050	0.18

Total variance is equal to one.

a Sex included in the model where *P* < 0.1.

b Square root of trait used to normalize the residuals. Bold indicates significant heritabilities (*P* <0.05).

For the vast majority of traits, the proportion of variance that can be attributed to additive genetic factors is considerably larger than the proportion that can be attributed to age, heteroplasmy, and sex suggesting that, in m.3243A>G carriers, nuclear background has a larger clinical effect than these known risk factors.

Estimates of the contribution of additive genetic factors to some of the defining features of MELAS, such as stroke‐like episodes and seizures, are particularly small. However, these traits are much lower in frequency (Fig. 1), reducing the ability of family‐based tests to measure heritability using a family‐based study design.

### Psychiatric involvement, migraine, and encephalopathy are heritable as binary traits

The variance component methods implemented in SOLAR assume normally distributed quantitative trait data. A limitation of our study is that the trait data are ordered categorical variables and do not all follow a normal distribution. To address this issue, we also calculated heritabilities considering the traits as binary variables (Table 3). The heritability estimate for psychiatric involvement remains significant (*h* ^2^ = 0.55, *P* = 0.0016), and for migraine (*h* ^2^ = 0.77, *P* = 0.0104) and encephalopathy (*h* ^2^ = 0.64, *P* = 0.0408), the estimates are greater for the binary trait. This may be due to a stricter definition of the phenotypes, with milder cases coded as unaffected in the binary model. Heritability estimates for cerebellar ataxia, cognition, and hearing impairment are no longer significant, but this is not surprising given the loss of information due to converting NMDAS scores to binary traits and may be due to incorrectly assigning milder individuals an unaffected status.

**Table 3:** Binary trait heritability (*h* ^2^)

Trait	Number of cases	*h* ^2^	SE	*P* value	Kullback‐Leibler *R* ^2^
Psychiatric involvement	**214**	**0.55**	**0.19**	**0.0016**	**0.01** **
Cerebellar ataxia	213	0.46	0.37	0.0957	0.19
Migraine	**214**	**0.77**	**0.33**	**0.0104**	**0.05** *
Cognition	205	0.46	0.91	0.1023	0.04
Dysphonia–dysarthria	213	0.13	0.53	0.4019	0.15
Encephalopathy	**214**	**0.64**	**0.36**	**0.0408**	**0.10**
Stroke‐like episodes	214	0.51	0.49	0.1423	0.12
Visual acuity	210	0.26	0.26	0.2130	0.04
Ptosis	213	0.15	0.39	0.3510	0.08*
CPEO	213	0.47	0.52	0.2102	0.06
Hearing impairment	214	0.52	0.38	0.0886	0.23*
Diabetes	214	0.37	0.35	0.1462	0.22
Cardiovascular involvement	193	0.08	0.36	0.4066	0.13

Converting NMDAS scores to a binary trait leads to a loss of information and, as a result, it was not possible to calculate heritabilities for neuropathy, seizures, gastrointestinal disturbance, or myopathy. Kullback‐Leibler *R* ^2^ represents the information gained by including heteroplasmy level and age (*and sex, where *P* < 0.1) in the model. **Psychiatric involvement was analyzed as a quasi‐quantitative trait to avoid numerical problems in SOLAR, and therefore, this figure represents the proportion of variance due to covariates. Bold indicates significant heritabilities (*P* < 0.05).

### Genetic correlation provides evidence for a shared genetic etiology

To determine the extent to which the heritability of traits is caused by shared genetic factors, a bivariate model was used to calculate the estimated genetic correlation between traits. There is significant genetic correlation between cerebellar ataxia and psychiatric involvement (rhoG ~ 0.70, SE = 0.22, *P* = 0.0081), cerebellar ataxia and migraine (rhoG ~ 0.72, SE = 0.32, *P* = 0.0312), encephalopathy and migraine (rhoG ~ 0.80, SE = 0.27, *P* = 0.0340), and cognition and hearing (rhoG ~ −0.61, SE = 0.25, *P* = 0.0170). This implies that these traits share a genetic etiology; that is, they may be influenced by variations in the same genes and is not surprising, given the role of the nervous system in these traits and the increasing evidence of pleiotropy in complex traits^31^, ^32^ Due to a reduction in power when using a bivariate model, we cannot exclude the possibility of shared genetic etiology between other pairs of traits.

## Discussion

The underlying cause of the extensive clinical heterogeneity associated with m.3243A>G‐related mitochondrial disease remains poorly understood. Given the heteroplasmic nature of this pathogenic variant and the progressive nature of disease, it was not unreasonable to hypothesize that heteroplasmy level and age explain some of this variance, although previous studies have only explained a small proportion.^12^, ^14^ In this study, we show that hearing impairment, diabetes, and cerebellar ataxia are the traits that are best explained by these factors, but pseudo‐*R* ^2^ values are low for all traits studied, ranging from 0.02 (psychiatric involvement, migraine, and gastrointestinal disturbance) to 0.17 (hearing impairment), leaving a considerable amount of variance unexplained. Notably, pseudo‐*R* ^2^ values for traits characteristic of the MELAS phenotype lie between 0.14 (encephalopathy) and 0.07 (stroke‐like episodes and seizures). Thus, predicting which patients are likely to develop severe disease using these risk factors is almost impossible.

Using a pedigree‐based study design, we have shown that additive genetic factors play a much larger role in determining the phenotypic outcome in m.3243A>G carriers than heteroplasmy level, age, and sex. Moderate to large estimates of heritability were observed for psychiatric involvement, cerebellar ataxia, migraine, cognition, and hearing impairment (*h* ^2^ range = 0.40–0.76). This is good evidence for the presence of nuclear factors influencing the clinical phenotype in m.3243A>G‐related disease. Pedigrees containing m.3243A>G carriers with similar phenotypic presentations have been previously described, suggesting the presence of a heritable factor influencing phenotype.^17^, ^33^ However, this is the first study to clearly demonstrate the presence of a nuclear effect in a whole cohort and to quantify this with heritability estimates.

We have confirmed the well‐recognized clinical heterogeneity associated with m.3243A>G using the NMDAS scale. As expected, features of the MELAS phenotype (seizures, encephalopathy, and stroke‐like episodes) are strongly correlated and a moderate correlation is seen between hearing and deafness, features of maternally inherited diabetes and deafness (MIDD).^7^, ^14^, ^30^, ^34^ Less anticipated were the moderate to strong correlations with cerebellar ataxia; notably for myopathy, dysphonia–dysarthria, and hearing impairment. This perhaps indicates overall neurodegeneration but may point toward a key role of the cerebellum in the pathology of m.3243A>G‐related disease, further supported by genetic correlations between cerebellar ataxia and psychiatric involvement and cerebellar ataxia and migraine, suggesting shared genetic etiology for these traits. Our observations are in‐line with previous reports describing prominent atrophy within the cerebellum of m.3243A>G patients both with and without stroke‐like episodes.^35^, ^36^ Whilst migraine and diabetes only correlate with a small number of other traits, suggesting that the underlying aetiologies of these traits are different and may be more heavily influenced by factors such as nuclear background and environmental factors, the correlation between migraine and gastrointestinal disturbance is intriguing. It is tempting to hypothesize that a similar biological mechanism may underlie these traits and could involve smooth muscle, given recent evidence that migraine‐associated genetic variants are enriched for genes involved in smooth muscle function.^37^ However, we should be cautious not to over‐interpret statistical correlations.

The correlations between psychiatric disturbance and seizures, encephalopathy, and stroke‐like episodes (*r* ~ 0.24–0.34) were also intriguing. Upon further investigation, we discovered that of the 22 patients in our cohort who have both encephalopathy and moderate to severe psychiatric disturbance (NMDAS≥3), 19 have evidence of encephalopathy documented before or within the same 12‐month period as the onset of these psychiatric symptoms. Therefore, psychosis may be linked to dysfunctional brain and such a hypothesis is supported by the well‐documented existence of postictal psychosis in epilepsy patients.^38^ We were not able to replicate the previously reported association between stroke‐like episodes and sex, although our data do show a similar trend.^14^ The over‐representation of males with stroke‐like episodes could be the result of ascertainment bias; we actively trace family members who carry the m.3243A>G variant resulting in the recruitment of more mildly affected females. However, the higher male penetrance for pathogenic mtDNA variants associated with Leber hereditary optic neuropathy is well documented and we do see a significant effect of sex for both ptosis and myopathy, supporting the role of sex in m.3243A>G‐related disease.^14^, ^39^

Although we estimate low heritabilities for stroke‐like episodes and seizures, we cannot exclude the possibility that nuclear factors are involved in these severe phenotypes. A number of our pedigrees were identified due to a diagnosis of MELAS in one family member, but many of these could not be recruited to the study due to the often fatal nature of MELAS, limiting our ability to measure heritability for these traits. Within this context, it is interesting to note that encephalopathy, a sign of substantial brain disease, is heritable when considered as a binary trait (*h* ^2^ = 0.64, *P* = 0.0408). Continued pedigree recruitment and long‐term follow‐up is needed to produce more accurate estimates.

The demonstration of nuclear background influencing clinical heterogeneity in m.3243A>G‐related disease has far‐reaching implications. First, it opens up the possibility of identifying these factors and, with rapid DNA sequencing technologies, ever‐expanding well‐characterized patient cohorts and pedigree tracing, this may be within our sights. Given the pedigrees that are available for study, areas of the nuclear genome responsible for these high heritabilities could be identified using genetic linkage analysis, which has the advantage of being able to detect effects due to rare and common variants, along with family‐specific effects.^40^ This would allow us to determine whether the nuclear effect is caused by many small effect loci, a small number with larger effect, or a combination of the two. Causal variants in these linked regions could be identified by next‐generation sequencing, and their role in pathogenesis confirmed using molecular biochemical techniques. Identifying interactions between nuclear variation and m.3243A>G should allow the development of more accurate tools for disease prognosis and progression, as well as having translational impact through the provision of accurate genetic advice to families considering reproductive options. Understanding these complex interactions and how they lead to disease is particularly pertinent given the recent development of pronuclear transfer (PNT) in the prevention of transmission of mtDNA mutations.^41^ Indeed, nuclear factors involved in m.3243A>G‐related disease may also impact other clinically heterogeneous mitochondrial diseases such as those caused by m.8344A>G and mutations within POLG.^42^, ^43^

We acknowledge that there are caveats to the interpretation of our data. Firstly, using easily sampled tissues such as blood or urine to detect the level of heteroplasmy does not truly represent the heteroplasmy levels present in tissues that are the most severely affected by m.3243A>G‐related disease. Although blood m.3243A>G heteroplasmy levels decline over time, age‐corrected blood heteroplasmy level is just as good as muscle level at predicting overall disease progression and is a better predictor than urine level, which is affected by sex and is more variable (unpublished data). The mechanisms involved in the establishment of the tissue distribution of heteroplasmy levels and mtDNA copy number levels are poorly understood, although nuclear factors may play a role and could represent one mechanism by which nuclear variation modifies phenotype.^17^

Second, we have used the NMDAS to define m.3243A>G‐related traits and this should be considered when interpreting our results. For instance, psychiatric disturbance can range from mild (e.g., reactive depression) to severe (e.g., bipolar disorder and psychosis), and is likely to represent a number of different conditions with different aetiologies. Similarly, the NMDAS migrainous question does not distinguish between different types of migrainous headache and impairment of visual acuity could represent a number of different pathologies, including pigmentary retinopathy and optic atrophy. Therefore, the underlying genetic architecture influencing these traits is also likely to be heterogeneous. In addition, the NMDAS scale for cognition primarily assesses processing speed and pre‐morbid IQ, known to decline in patients with mitochondrial disease, and we have not corrected this for confounders such as educational attainment. However, the NMDAS is clinically validated and widely used and is the only currently available tool for this type of analysis. In further support of its use, our heritability estimates fall within population‐based estimates for migraine (28–65%),^44^ age‐related sensory hearing loss (25–55%)^45^, and cognition (28–62%, in patients with type 2 diabetes).^46^ Population‐based heritability estimates for major depression are 31–42%.^47^ Our estimate for psychiatric disturbance in m3243A>G‐related disease is larger than this; however, estimates are higher for more severe psychiatric disorders such as schizophrenia (64–67%) and bipolar disorder (59–62%), consistent with our results.^48^, ^49^

Finally, although the variance component methods implemented in SOLAR estimate the proportion of variance due to additive genetic factors using the degree of relatedness between individuals, heritability estimates may be inflated due to increased shared environment between more closely related individuals. Even with high heritabilities, the nuclear genetic factors may not be easy to identify. Our patient cohort is large and well characterized for such a rare disease, but sample size could be a limitation. Most genetic associations in common disease confer a relatively small effect on phenotype with odds ratios of 1.2–1.5, their detection requiring large sample sizes.^50^ However, there are exceptions to this, for example, polymorphisms in *CFH* contributing to age‐related macular degeneration, and rare variants with higher penetrance and could be detected using a family‐based approach.^51^

This study identifies nuclear variation as a key contributor to the vast clinical heterogeneity seen in m.3243A>G‐related disease, opening up the possibility of identifying these factors and improving our understanding of this complex mitochondrial disease. It also highlights the benefits of pedigree tracing, not only to improve patient care and the rate of diagnosis but also to better understand disease etiology. The molecular mechanisms underlying m.3243A>G‐associated traits are likely to involve a complex interplay between m.3243A>G mutant load, mtDNA copy number, nuclear genetic factors, epigenetics, and environmental exposures. The next challenge is to identify these nuclear risk factors in order to better understand the cellular and tissue‐specific effects in m.3243A>G‐related disease and provide improved estimates of disease prognosis and counseling to patients.

## Conflict of interests

We declare no conflict of interests.

## Author Contributions

Conception and design of the study: SJP, JPG, RMcF, RWT, DMT, HJC, IJW; Acquisition and analysis of data: SJP, JPG, YSN, AMS, GSG; Drafting the manuscript: SJP; Critical review of the manuscript: RWT, JPG, YSN, DMT, HJC, IJW, GSG, RMcF.

## References

[1] Alston CL , Rocha MC , Lax NZ , et al. The genetics and pathology of mitochondrial disease. J Pathol 2017;241(2):236–250. 27659608 10.1002/path.4809 PMC5215404

[2] Calvo SE , Clauser KR , Mootha VK . MitoCarta2.0: an updated inventory of mammalian mitochondrial proteins. Nucleic Acids Res 2016;44(D1):D1251–D1257. 26450961 10.1093/nar/gkv1003 PMC4702768

[3] Gorman GS , Chinnery PF , DiMauro S , et al. Mitochondrial diseases. Nat Rev Dis Primers 2016;20(2):16080. 10.1038/nrdp.2016.80 27775730

[4] Goto Y , Nonaka I , Horai S . A mutation in the tRNA(Leu)(UUR) gene associated with the MELAS subgroup of mitochondrial encephalomyopathies. Nature 1990;348(6302):651–653. 2102678 10.1038/348651a0

[5] Elliott HR , Samuels DC , Eden JA , et al. Pathogenic mitochondrial DNA mutations are common in the general population. Am J Hum Genet 2008;83(2):254–260. 18674747 10.1016/j.ajhg.2008.07.004 PMC2495064

[6] Gorman GS , Schaefer AM , Ng Y , et al. Prevalence of nuclear and mitochondrial DNA mutations related to adult mitochondrial disease. Ann Neurol 2015;77(5):753–759. 25652200 10.1002/ana.24362 PMC4737121

[7] Nesbitt V , Pitceathly RD , Turnbull DM , et al. The UK MRC Mitochondrial Disease Patient Cohort Study: clinical phenotypes associated with the m.3243A>G mutation–implications for diagnosis and management. J Neurol Neurosurg Psychiatry 2013;84(8):936–938. 23355809 10.1136/jnnp-2012-303528

[8] Manwaring N , Jones MM , Wang JJ , et al. Population prevalence of the MELAS A3243G mutation. Mitochondrion 2007;7(3):230–233. 17300999 10.1016/j.mito.2006.12.004

[9] Sue CM , Quigley A , Katsabanis S , et al. Detection of MELAS A3243G point mutation in muscle, blood and hair follicles. J Neurol Sci 1998;161(1):36–39. 9879679 10.1016/s0022-510x(98)00179-8

[10] Rahman S , Poulton J , Marchington D , Suomalainen A . Decrease of 3243 A–>G mtDNA mutation from blood in MELAS syndrome: a longitudinal study. Am J Hum Genet 2001;68(1):238–240. 11085913 10.1086/316930 PMC1234919

[11] Rajasimha HK , Chinnery PF , Samuels DC . Selection against pathogenic mtDNA mutations in a stem cell population leads to the loss of the 3243A–>G mutation in blood. Am J Hum Genet 2008;82(2):333–343. 18252214 10.1016/j.ajhg.2007.10.007 PMC2427290

[12] Chinnery PF , Howell N , Lightowlers RN , Turnbull DM . Molecular pathology of MELAS and MERRF. The relationship between mutation load and clinical phenotypes. Brain. 1997 120(Pt 10):1713–1721. 9365365 10.1093/brain/120.10.1713

[13] Betts J , Jaros E , Perry RH , et al. Molecular neuropathology of MELAS: level of heteroplasmy in individual neurones and evidence of extensive vascular involvement. Neuropathol Appl Neurobiol 2006;32(4):359–373. 16866982 10.1111/j.1365-2990.2006.00731.x

[14] Mancuso M , Orsucci D , Angelini C , et al. The m.3243A>G mitochondrial DNA mutation and related phenotypes. A matter of gender? J Neurol 2014;261(3):504–510. 24375076 10.1007/s00415-013-7225-3

[15] Grady JP , Campbell G , Ratnaike T , et al. Disease progression in patients with single, large‐scale mitochondrial DNA deletions. Brain. 2014 137(Pt 2):323–334. 24277717 10.1093/brain/awt321 PMC3914470

[16] Ross JM , Stewart JB , Hagstrom E , et al. Germline mitochondrial DNA mutations aggravate ageing and can impair brain development. Nature 2013;501(7467):412–415. 23965628 10.1038/nature12474 PMC3820420

[17] Maeda K , Kawai H , Sanada M , et al. Clinical phenotype and segregation of mitochondrial 3243A>G mutation in 2 pairs of monozygotic twins. JAMA Neurol. 2016;73:990–993. 27323007 10.1001/jamaneurol.2016.0886

[18] Hudson G , Keers S , Yu Wai Man P , et al. Identification of an X‐chromosomal locus and haplotype modulating the phenotype of a mitochondrial DNA disorder. Am J Hum Genet 2005;77(6):1086–1091. 16380918 10.1086/498176 PMC1285165

[19] Battersby BJ , Loredo‐Osti JC , Shoubridge EA . Nuclear genetic control of mitochondrial DNA segregation. Nat Genet 2003;33(2):183–186. 12539044 10.1038/ng1073

[20] Visscher PM , Hill WG , Wray NR . Heritability in the genomics era–concepts and misconceptions. Nat Rev Genet 2008;9(4):255–266. 18319743 10.1038/nrg2322

[21] Schaefer AM , Phoenix C , Elson JL , et al. Mitochondrial disease in adults: a scale to monitor progression and treatment. Neurology 2006;66(12):1932–1934. 16801664 10.1212/01.wnl.0000219759.72195.41

[22] deLaat P , Janssen MC , Alston CL , et al. Three families with ‘de novo’ m.3243A > G mutation. BBA Clin. 2016;6:19–24. 27331024 10.1016/j.bbacli.2016.04.007 PMC4900294

[23] R Core Team . R: a language and environment for statistical computing. R Foundation for Statistical Computing, Vienna, Austria URL https://www.R-project.org/ 2016.

[24] Harrell FE Jr . Hmisc: Harrell Miscellaneous. R package version 4.0‐0. https://CRAN.R-project.org/package=Hmisc2016.

[25] Christensen RHB . ordinal ‐ Regression Models for Ordinal Data. R package version 2015.6‐28. http://www.cran.r-project.org/package=ordinal/2015.

[26] McFadden D . The measurement of urban travel demand. J Public Economics 1974;3:303–328.

[27] Perneger TV . What's wrong with Bonferroni adjustments. BMJ 1998;316(7139):1236–1238. 9553006 10.1136/bmj.316.7139.1236 PMC1112991

[28] Almasy L , Blangero J . Multipoint quantitative‐trait linkage analysis in general pedigrees. Am J Hum Genet 1998;62(5):1198–1211. 9545414 10.1086/301844 PMC1377101

[29] Almasy L , Dyer TD , Blangero J . Bivariate quantitative trait linkage analysis: pleiotropy versus co‐incident linkages. Genet Epidemiol 1997;14(6):953–958. 9433606 10.1002/(SICI)1098-2272(1997)14:6<953::AID-GEPI65>3.0.CO;2-K

[30] van den Ouweland JM , Lemkes HH , Ruitenbeek W , et al. Mutation in mitochondrial tRNA(Leu)(UUR) gene in a large pedigree with maternally transmitted type II diabetes mellitus and deafness. Nat Genet 1992;1(5):368–371. 1284550 10.1038/ng0892-368

[31] Pickrell JK , Berisa T , Liu JZ , et al. Detection and interpretation of shared genetic influences on 42 human traits. Nat Genet 2016;48(7):709–717. 27182965 10.1038/ng.3570 PMC5207801

[32] Bulik‐Sullivan B , Finucane HK , Anttila V , et al. An atlas of genetic correlations across human diseases and traits. Nat Genet 2015;47(11):1236–1241. 26414676 10.1038/ng.3406 PMC4797329

[33] Koga Y , Akita Y , Takane N , et al. Heterogeneous presentation in A3243G mutation in the mitochondrial tRNA(Leu(UUR)) gene. Arch Dis Child 2000;82(5):407–411. 10799437 10.1136/adc.82.5.407 PMC1718342

[34] de Laat P , Koene S , van den Heuvel LP , et al. Clinical features and heteroplasmy in blood, urine and saliva in 34 Dutch families carrying the m.3243A > G mutation. J Inherit Metab Dis 2012;35(6):1059–1069. 22403016 10.1007/s10545-012-9465-2 PMC3470685

[35] Fromont I , Nicoli F , Valero R , et al. Brain anomalies in maternally inherited diabetes and deafness syndrome. J Neurol 2009;256(10):1696–1704. 19536585 10.1007/s00415-009-5185-4

[36] Tschampa HJ , Urbach H , Greschus S , et al. Neuroimaging characteristics in mitochondrial encephalopathies associated with the m.3243A>G MTTL1 mutation. J Neurol 2013;260(4):1071–1080. 23196335 10.1007/s00415-012-6763-4

[37] Gormley P , Anttila V , Winsvold BS , et al. Meta‐analysis of 375,000 individuals identifies 38 susceptibility loci for migraine. Nat Genet 2016;48(8):856–866. 27322543 10.1038/ng.3598 PMC5331903

[38] Kanner AM , Rivas‐Grajales AM . Psychosis of epilepsy: a multifaceted neuropsychiatric disorder. CNS Spectr 2016;21(3):247–257. 27322691 10.1017/S1092852916000250

[39] Meyerson C , Van Stavern G , McClelland C . Leber hereditary optic neuropathy: current perspectives. Clin Ophthalmol 2015;9:1165–1176. 26170609 10.2147/OPTH.S62021 PMC4492634

[40] Ott J , Wang J , Leal SM . Genetic linkage analysis in the age of whole‐genome sequencing. Nat Rev Genet 2015;16(5):275–284. 25824869 10.1038/nrg3908 PMC4440411

[41] Hyslop LA , Blakeley P , Craven L , et al. Towards clinical application of pronuclear transfer to prevent mitochondrial DNA disease. Nature 2016;534(7607):383–386. 27281217 10.1038/nature18303 PMC5131843

[42] Altmann J , Buchner B , Nadaj‐Pakleza A , et al. Expanded phenotypic spectrum of the m.8344A>G “MERRF” mutation: data from the German mitoNET registry. J Neurol 2016;263(5):961–972. 26995359 10.1007/s00415-016-8086-3

[43] Rajakulendran S , Pitceathly RD , Taanman JW , et al. A clinical, neuropathological and genetic study of homozygous A467T POLG‐related mitochondrial disease. PLoS ONE 2016;11(1):e0145500. 26735972 10.1371/journal.pone.0145500 PMC4703200

[44] Gardner KL . Genetics of migraine: an update. Headache 2006;46(Suppl 1):S19–S24. 16927960 10.1111/j.1526-4610.2006.00486.x

[45] Gates GA , Couropmitree NN , Myers RH . Genetic associations in age‐related hearing thresholds. Arch Otolaryngol Head Neck Surg 1999;125(6):654–659. 10367922 10.1001/archotol.125.6.654

[46] Cox AJ , Hugenschmidt CE , Raffield LM , et al. Heritability and genetic association analysis of cognition in the Diabetes Heart Study. Neurobiol Aging 2014;35(8):1958. e3‐ e12. 10.1016/j.neurobiolaging.2014.03.005 PMC4418030 24684796

[47] Sullivan PF , Neale MC , Kendler KS . Genetic epidemiology of major depression: review and meta‐analysis. Am J Psychiatry 2000;157(10):1552–1562. 11007705 10.1176/appi.ajp.157.10.1552

[48] Wray NR , Gottesman II . Using summary data from the danish national registers to estimate heritabilities for schizophrenia, bipolar disorder, and major depressive disorder. Front Genet 2012;3:118. 22783273 10.3389/fgene.2012.00118 PMC3387670

[49] Lichtenstein P , Yip BH , Bjork C , et al. Common genetic determinants of schizophrenia and bipolar disorder in Swedish families: a population‐based study. Lancet 2009;373(9659):234–239. 19150704 10.1016/S0140-6736(09)60072-6 PMC3879718

[50] Manolio TA , Collins FS , Cox NJ , et al. Finding the missing heritability of complex diseases. Nature 2009;461(7265):747–753. 19812666 10.1038/nature08494 PMC2831613

[51] Klein RJ , Zeiss C , Chew EY , et al. Complement factor H polymorphism in age‐related macular degeneration. Science 2005;308(5720):385–389. 15761122 10.1126/science.1109557 PMC1512523

